# Synchronisation in the beta frequency-band — The bad boy of parkinsonism or an innocent bystander?

**DOI:** 10.1016/j.expneurol.2009.02.003

**Published:** 2009-05

**Authors:** Alexandre Eusebio, Peter Brown

**Affiliations:** a Department of Neurology and Movement Disorders, CHU Timone, Marseille, France; b Sobell Department of Motor Neuroscience and Movement Disorders, Institute of Neurology, 33 Queen Square, London, WCIN 3BG, UK.

## Abstract

Excessive synchronisation of basal ganglia neuronal activity in the beta frequency band has been implicated in Parkinson's disease. In a recent issue of Experimental Neurology, Bronte-Stewart, H., Barberini, C., Koop, M.M., Hill, B.C., Henderson, J.M., Wingeier, B., 2009. The STN beta-band profile in Parkinson's disease is stationary and shows prolonged attenuation after deep brain stimulation. Exp. Neurol. 215, 20–28. demonstrate that such activity is consistent over time and provide further evidence that deep brain stimulation is associated with its suppression. However, the extent to which beta synchrony has a mechanistic (rather than epiphenomenal) role in parkinsonism remains unclear, and the suppression of this activity by deep brain stimulation is contentious. This commentary discusses the evidence for and against a role for excessive beta synchrony in mediating the parkinsonian phenotype and in providing a possible mechanism to explain the therapeutic effects of deep brain stimulation in Parkinson's disease.

What goes wrong to cause motor impairment in Parkinson's disease? One way to answer this is to record neuronal activity directly from the basal ganglia of afflicted patients or of animals deliberately made parkinsonian through the use of selective toxins. The last few decades have seen blame shift from altered discharge rates to altered discharge patterns and now to excessive synchronisation of activity across populations of neurons. The latter leads to summation of electrical fields which can be picked up as undulations of the local field potential and may even be recorded through the macroelectrodes used for therapeutic deep brain stimulation (DBS). The paper by Bronte-Stewart et al. (2009) adds to the growing evidence that excessive and oscillatory synchronisation occurs in the basal ganglia in Parkinson's disease and that this has a predilection for the beta frequency band centred around 20 Hz. Bronte-Stewart and colleagues recorded local field potentials from the subthalamic nucleus in patients undergoing surgery for deep brain stimulation of this target, an effective treatment of Parkinson's disease and demonstrated that oscillatory activity was prominent in the beta band, and that this was a consistent phenomenon over many minutes of recording. Having established this they went on to explore whether deep brain stimulation might act by suppressing this pathological activity.

Herein lays a problem. Therapeutic DBS is performed at very high frequencies (> 100 Hz) and at voltages that are huge compared to the spontaneous beta activity in the local field potential. Accordingly, it has proven very difficult to derive useful field potential recordings during DBS. All is not lost, however, as many patients with Parkinson's disease experience clinical benefit that outlasts the period of DBS by seconds to minutes. Bronte-Stewart and colleagues cleverly recorded during this period, with the expectation that beta activity might be suppressed (Wingeier et al., 2006), and in their current paper they develop this line, demonstrating that suppression occurs and its duration can be extended by longer periods of DBS. Although the level of beta suppression was not correlated with clinical state in their study due to the practical constraints of recordings being made in the operating theatre, their findings are complemented by those in another recent report, where suppression of beta activity following cessation of DBS was also demonstrated to correlate with the degree of residual motor improvement (Kühn et al., 2008).

Nevertheless, these findings remain contentious, with a variety of mechanisms being proffered to explain the beneficial effects of DBS (Hammond et al., 2008). The group of Priori in Milan have, in particular, questioned whether DBS works by suppressing beta synchrony. They failed to find any consistent suppression in beta activity after the cessation of DBS (Priori et al., 2006). It might be possible to dismiss their findings on the basis that they averaged local field potential activity over too long a period following cessation of DBS, as argued by Bronte-Stewart et al. (2009), but for the results of their follow-up study. In the latter, they, through a feat of amplifier design (Rossi et al., 2007), were able to record local field potential activity actually during DBS. Overall, they found no suppression of beta activity, but not all their patients had a peak in this frequency band at baseline (Rossi et al., 2009), so that DBS could not suppress beta any further. One reason for the absence of a baseline beta peak might be that half their patients were recorded on levodopa, which is known to suppress beta activity (Hammond et al., 2007), another may be that some of their patients had diminished beta activity associated with the temporary ‘stun’ effect that may be seen after surgery, and which is discussed later.

The effect of DBS on beta activity should be seen as part of a wider debate as to whether beta synchrony mediates parkinsonism. Several reports have shown a correlation between the levodopa-induced suppression of beta activity and levodopa-induced improvement in bradykinesia and rigidity (Kühn et al., 2006; Weinberger et al., 2006; Ray et al., 2008; Kühn et al., 2008), and yet these only provide evidence by association. There have been additional attempts to show a more causal link by directly stimulating the subthalamic nucleus at 20 Hz instead of the more usual therapeutic frequencies (> 100 Hz) in parkinsonian patients chronically implanted with DBS electrodes (Chen et al., 2007; Eusebio et al., 2008). Although these confirm the predicted slowing of motor performance, the effects are small and of the order of about a 10% slowing in finger tapping.

The relatively modest effect of direct stimulation may be ascribed to various factors: the failure to mimic the pathological state through the delivery of only one synchronising discharge per cycle of the 20 Hz rhythm, a ceiling effect of beta synchrony, given that tested patients have had advanced Parkinson's disease and have been withdrawn overnight from their dopaminergic therapy, or a single site intervention in an extended loop that ordinarily shows excessive beta synchrony at all sampled levels (Brown et al., 2001; Williams et al., 2002; Lalo et al., 2008). But the modest effects also raise the possibility that the bulk of the parkinsonian state is not mediated by beta synchrony at all. Certainly, there is now general consensus that this activity is not causally linked to parkinsonian tremor (Amirnovin et al., 2004; Kühn et al., 2006; Weinberger et al., 2006; Ray et al., 2008; Kühn et al., 2009). In addition, there is no correlation between beta synchrony in the subthalamic nucleus and the UPDRS motor score recorded off dopaminergic therapy (Weinberger et al., 2006; Ray et al., 2008). So, at best, beta synchrony seems to relate to that portion of the bradykinetic-rigid state that can be reversed by dopaminergic therapy and that is therefore likely to improve with subthalamic nucleus DBS (Charles et al., 2002). As Parkinson's disease becomes more advanced, therapy induced reversal of parkinsonism is incomplete so that even on combined drug therapy and subthalamic nucleus DBS, United Parkinsons Disease Rating Scale (UPDRS) motor scores are generally around 15–20 in chronically stimulated patients (Rodriguez-Oroz et al., 2005). At the other end of the scale, it is also uncertain whether beta synchrony relates to very early parkinsonian motor impairment. As patients at this stage do not undergo functional neurosurgery, recordings of synchronised activity have to be derived from animal models of parkinsonism and these suggest that the onset of prominent synchrony is delayed, arguably beyond the appearance of the very first motor deficits (Leblois et al., 2007; Mallet et al., 2008a).

Another possible argument against a mechanistic role for beta synchrony in parkinsonism is the fact that a minority of patients (none in the paper of Bronte-Stewart et al. 2009) have very little beta power in their subthalamic local field potentials when recorded several days after implantation, in the interval between the latter and further surgery to introduce and connect a subcutaneous battery and stimulator. Our experience is that such patients have prominent stun effects, in other words temporary post-operative amelioration of parkinsonism even before stimulation and which often makes the full efficacy of DBS difficult to establish during the immediate post-operative period. The stun effect is believed to be due to local trauma and oedema, and there is evidence to link intra-operative stun effects with partial beta suppression (Chen et al., 2006a). Nevertheless, no study has explicitly sought a relationship between the post-operative stun effect and loss of beta synchrony.

How might excessive neuronal synchronisation in the beta band compromise motor processing leading to motor impairment? A recent study in an animal model of parkinsonism in which there is also pathological beta activity has explored this (Mallet et al., 2008a). The study examined the mutual information between subthalamic nucleus neuronal ensembles in 6-hydroxy-dopamine midbrain lesioned rodents and found this to be significantly increased as compared to control animals. In this instance mutual information implies that information channels are being squandered on the same message, so that the capacity for information encoding and therefore processing across the whole system becomes smaller in parkinsonian animals. But how can this be reconciled with another recent report that subthalamic nucleus DBS using therapeutic parameters leads to a severe reduction in entropy and hence information coding capacity in single neurons in the pallidum and thalamus (Dorval et al., 2008)? Maybe the solution can be found in the old ‘noisy signal’ hypothesis (see Brown and Eusebio, 2008). The implication is that in the parkinsonian state only partial processing is possible in the basal ganglia and this kind of activity effectively acts as a disruptive ‘noisy signal’ and is worse than a fixed and unfamiliar patterning of activity when passed on to other processing units like the cortex. High frequency DBS acts to override this functionally deleterious partial processing, replacing it with a highly organised, very low entropy, discharge pattern which mirrors the DBS regimen and is less disruptive to other sites. In other words high frequency DBS replaces a noisy signal with a less disruptive one. Whether the new pattern is innocuous or just not quite as bad is another question (Chen et al., 2006b).

Another unresolved question is whether there is something especially important about certain frequencies of pathologically synchronised oscillation or whether it is the oscillatory synchronisation per se, rather than the precise frequency that is more relevant. Most patients show evidence of synchronisation in the beta frequency band, but this tells us more about the resonance frequencies of circuits in the absence of dopaminergic input than whether synchronisation with a lower or higher frequency might be just as pathogenic if it were to occur. Indeed, one report suggests that within certain limits (8–35 Hz), changes in synchronisation rather than frequency correlate better with levodopa-induced improvement in bradykinesia and rigidity (Kühn et al., 2009). At even higher frequencies, however, there seems to be no antikinetic effect, but rather a possible favouring of movement (Brown, 2003).

Finally, the very existence of DBS after-effects on beta-activity, as well as the intriguing observation that a longer duration of DBS leads to a longer period of beta suppression, deserves further comment. Effects that persist over tens of seconds or longer suggest that the overriding of pathological patterning by DBS may be insufficient to explain all responses to this treatment and implies the existence of plasticity phenomena in the basal ganglia – cortical network. Relevant in this regard is the evidence of synaptic plasticity occurring in cortico-striatal transmission during high-frequency stimulation of the subthalamic nucleus in a rat model of Parkinson's disease (Gubellini et al., 2006). Yet, not all patients experience clinical after-effects after DBS, and in those that do there is a progressive and sequential return of different symptoms after the discontinuation of stimulation (Temperli et al., 2003). Together these observations argue for a variety of pathophysiological mechanisms in Parkinson's disease, which differentially contribute to separate parkinsonian features.

In summary, beta synchrony may relate to some but not all elements of motor impairment in Parkinson's disease, and the jury is still out on its quantitative importance and the means by which it might disturb motor processing. One thing that seems reasonably clear, however, is that beta synchrony is a good biomarker of the established akinetic-rigid state in both patients (Hammond et al., 2007) and many animal models of parkinsonism (Sharott et al., 2005; Costa et al., 2006; Mallet et al., 2008a,b). As such it may be a useful surrogate marker in pharmacological studies and in closed loop DBS protocols. In the latter case, detection of beta activity in the subthalamic local field potential could be used to drive feedback control of stimulation, where the primary purpose of this is to treat bradykinesia and rigidity, rather than tremor. This could save on battery life, limit side-effects and also save programming time Jensen et al., 2008. The paper by Bronte-Stewart and colleagues adds to the growing evidence that beta synchrony is a consistent feature of parkinsonism and suppressed by DBS, helping pave the way for closed loop stimulation in the future.
